# Characteristics of petroleum contaminants and their distribution in Lake Taihu, China

**DOI:** 10.1186/1752-153X-6-92

**Published:** 2012-08-31

**Authors:** Jixiang Guo, Jia Fang, Jingjing Cao

**Affiliations:** 1 Enhanced Oil Recovery Institute, China University of Petroleum, Beijing, 102249, Peoples Republic of China; 2 Tianjin Chen Li Technology Corporation, Ltd, Tianjin, 300400, People Republic of China

**Keywords:** Petroleum contaminants, Distribution, n-alkanes, PAHs, GC/MS, Taihu Lake

## Abstract

**Background:**

Taihu Lake is a typical plain eutrophic shallow lake. With rapidly economic development of the lake area, the petroleum products and oil wastewater produced in various processes have been inevitably discharged into Taihu Lake. As the major fresh water resource in the economically developed region of Yangtze River Delta, the water quality and environmental condition of Taihu Lake have the direct bearing on the natural environment and sustainable development of economy in this region. For this reason we carried out the study to explore the composition, distribution characteristics and sources of petroleum contaminants in Taihu Lake. The aim of this study was to provide the basis for standard management and pollution control of the Taihu Lake environment.

**Results:**

The result showed that water samples from near industrial locations were of relatively higher petroleum contaminants concentrations. The oil pollutants concentrations in different areas of Lake Taihu ranged from 0.106 mg/L to 1.168 mg/L, and the sequence of total contents distribution characteristics of petroleum pollutants from high to low in different regions of Taihu Lake was: “Dapu”, “Xiaomeikou”, “Zhushan Bay”, “Lake center”, “Qidu”. The results showed that total concentrations of n-alkanes and PAHs ranged from 0.045 to 0.281 mg/L and from 0.011 to 0.034 mg/L respectively. In the same region, the concentrations of hydrocarbon pollutants in the surface and bottom of the lake were higher than that in the middle.

**Conclusion:**

This paper reached a conclusion that the petroleum contaminants in Taihu Lake mainly derived from petroleum pollution caused by human activities as indicated by OEP, bimodal distribution, CPI, Pr/Ph ratio, the LMW/HMW ratio and other evaluation indices for sources of n-alkanes and polycyclic aromatic hydrocarbons (PAHs).

## Introduction

Taihu Lake is one of the five largest freshwater lakes in China, and it is a typical plain eutrophic shallow lake. As the major fresh water resource in the economically developed region of Yangtze River Delta, the water quality and environmental condition of Taihu Lake have direct influence on the natural environment and sustainable development of economy in this region [1]. With rapidly economic development of the lake area, the petroleum products and oil wastewater produced in the process of oil processing, transportation and application of various refined oil have been inevitably discharged into Taihu Lake.

Petroleum is a complex mixture mainly composed of hydrocarbons, in which most of alkanes are proved to be narcotic and irritant, and most of PAHs have strong toxicity, carcinogenicity, teratogenicity and mutagenicity [2,3]. The large amount of oil pollutants in water environment will cause serious pollution to the water ecosystems and direct harm to the health of the living creatures and human bodies [4].

The content of petroleum pollutants is one of the parameters evaluating the water quality and its impact is increasingly important [5]. At present, the research on petroleum pollutants in Taihu is rarely reported, and most of the reports mainly focused on the analysis of PAHs in surface sediments of Taihu Lake [6-8]. Few studies have been reported on petroleum contaminants in the whole water system of Taihu Lake. Taking Taihu Lake, which is one of the five largest lakes in China [9], as the research object, this paper systematically studied the composition, distribution characteristics and sources of petroleum pollutants in Taihu Lake by means of infrared spectrophotometry stipulated in Surface Water Quality Standards (GB/T 16488–1996 in China) [10] and gas chromatography–mass spectrometry in order to provide basic information for standard management and pollution control of the lake environment.

## Experimental methods

### Instruments and reagents

#### Instruments

Agilent 7890A/5975C GC/MS; OIL-20A infrared luminosity analyzer; rotary evaporator; separatory funnel; glass adsorption column.

#### Reagents

Carbon tetrachloride (analytical pure).

Standard indicators (all chromatographically pure): internal standard indicators: deuterated n-tetracosane (0.15 μg/μL);deuterated dibenzothiophene (0.103 μg/μL);recovery indicators: 5α-androstane (1.00 mg/mL);deuterated acenaphthene (0.40 mg/mL). Anhydrous sodium sulfate (heated for 2 hours under 300°C and cooled down to room temperature), sodium chloride, dichloromethane (all analytical pure).

### Sampling and analysis

#### Collection of water samples

**Figure 1:**
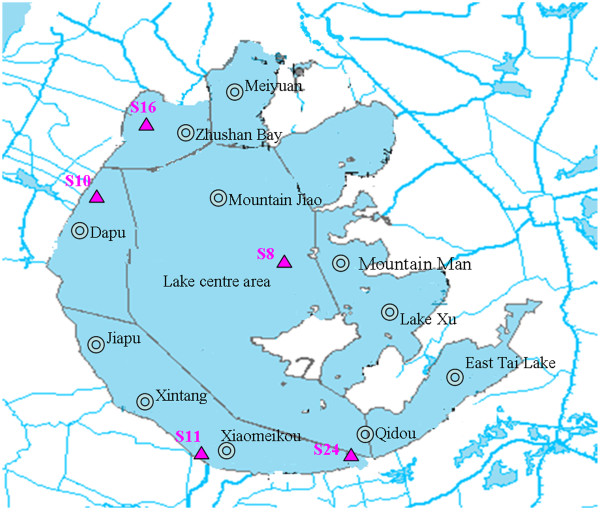
**The distribution of sampling sites in Taihu Lake.** The distribution of sampling sites in Taihu Lake is shown in Figure 1, the sampling sites were S8: Lake center, S10: Dapu, S11: Xiaomeikou, S16: Zhushan Bay, and S24: Qidu. The water samples from different water levels in the sampling site S16 were also collected: S16-1: 1 m below the water surface, S16-2: 2 m below the water surface of S16.

Water samples were collected and preserved according to the methods of surface water and wastewater monitoring specifications (HJ/T 91–2002, China) [11]. Five representative sampling points in the whole region of Taihu Lake were selected. The distribution of sampling sites in Lake Taihu is shown in Figure 1.

#### Pretreatment of water samples

The recovery indicators of 5α-androstane and deuterated acenaphthene were added into the water sample before extracting and concentrating [12]. Water sample of specific volume was poured into a 1000 mL separatory funnel. 50 mL dichloromethane was added into the sampling bottle, and the sample bottle was shaken for 30 s to wash its inner surface. The solvent was then transferred into the separatory funnel, which then stand still for layering after a full vibration about 3 minutes. And then dichloromethane of 20 mL were used to extract the sample twice [13]. Na_2_SO_4_ was added to the extract liquid for removing traces of water. Finally, the extract liquid in magnesium silicate column was concentrate to 5 mL by use of a rotary evaporator and nitrogen purge.

#### Determination of the total content of petroleum contaminants

The total content of petroleum contaminants in Taihu Lake was determined by infrared spectrophotometry stipulated in Water quality determination of petroleum oil, animal and and vegetable oils-Infrared photometric method (GB/T 16488–1996 in China) [10].

OIL-20A infrared luminosity analyzer was adopted in the determination of the total content of petroleum contaminants. The principle of the analyzer is nondispersive infrared analysis*.*

After pretreatment, the filter liquor after adsorbed by magnesium silicate column was measured by OIL-20A infrared luminosity analyzer. The absorption of the filter liquid was measured, then the analyzer converted the absorption into the total content of petroleum contaminants.

#### GC/MS analysis

The internal standards n-C_24_D_50_ and deuterated dibenzothiophene of n-alkanes and PAHs were added respectively into the concentrated liquid; finally the concentrated liquid was separated by GC/MS and analyzed quantitatively by internal standard method.

The column used was HP-5MS flexible quartz capillary (60 m× 0.25 mm× 0.25 μm). The column was held at 50°C for 1 min, heated to 300°C at a rate of 10°C/min, and held for 20 min. Helium was used as the carrier gas, and the flow rate was maintained at 1 mL/min. The splitting ratio was 50/1 and the temperature of injection port was 290°C. The holding time of solvent was 4 min. The mass range scanned was from 35 to 500 amu under full scan acquisition mode.

The qualitative analysis browser – Qual Browser was adopted to analyze the chromatograms obtained by GC/MS method. The chormatograms were intergrated after being activated, then the compositions and relative contents of petroleum contaminants in the water samples were calculated using the square penks normalizaion of the intergral regions.

## Results and discussion

### Determination of total content of petroleum contaminants

Table 1 shows the concentrations of petroleum contaminants in water samples from different sampling points of Taihu Lake. The distribution of petroleum contaminants in the surface water of Taihu Lake is characterized by the sequence S10 (Dapu)>S11 (Xiaomeikou)>S16 (Zhushan Bay)>S8 (Lake center)>S24 (Qidu). The range of the total concentration (average concentration) was from 0.106 mg/L to 1.168 mg/L and the mean value was 0.572 mg/L.

**Table 1:** The content of total petroleum contaminants detected by infrared photometric method (unit: mg/L)

**Sampling points**	**S8**	**S10**	**S11**	**S24**	**S16**	**S16 -1**	**S16-2**
**Concentration**	0.380	1.150	0.640	0.106	0.578	0.210	1.572
	0.370	1.116	0.712	0.110	0.448	0.248	1.590
	0.372	1.238	0.718	0.101	0.546	0.232	1.656
Average concentration	0.374	1.168	0.690	0.106	0.524	0.230	1.606
**RSD**(%)	1.41	5.39	6.29	4.37	12.93	8.30	2.75
**Recovery** (%)	92.10	86.85	75.50	107.16	83.30	101.40	111.20

Note:

S8:Lake center; S10: Dapu; S11:Xiaomeikou; S24: Qidu; S16: Zhushan Bay; S16-1: 1 m below the water surface of S16; S16-2: 2 m below the water surface of S16 (the bottom of S16).

A series of parallel tests (three times for each sample) were carried out to determine the contents of the total petroleum contaminants.The average content of total petroleum contaminants in different water samples were calculated, and the range is from 0.106 mg/L to 1.168 mg/L.

Yili River system in the west side of Taihu flows into Taihu near Dapukou, and the Yili River region belongs to a region with relatively more developed economy, and the stream of Yili River carries pollutants with high concentrations discharged from industrial and living areas in the region into the Taihu Lake, which causes the concentration of oil pollutants in Dapu of Taihu Lake to be the highest of all.

As there are fewer human activities in East Taihu Lake area, the lake area has a relatively better water quality, and the experimental results also show that the concentration of oil pollutants in Qidu is the lowest.

Zhushan Bay is one of the most seriously eutrophicated areas in Taihu Lake, and its water pollution is relatively more serious with the content of oil pollutants exceeding the limit value of oil Class IV in Surface Water Quality Standards [14]. After a period of volatilization, diffusion and degradation of the oil pollutants in the lake, the content of oil pollutants at the central lake is relatively lower than that at each entrance of the lake. At different water levels of the same sampling site (S16, S16-1, S16-2), the highest concentration of petroleum contaminants was in the bottom layer (S16-2), followed by that in the surface layer (S16), and that in the middle layer (S16-1) was the lowest. That is closely related with the properties of petroleum such as its high volatility, light density, high diffusivity and so on. The bottom layer has the highest concentration of petroleum hydrocarbons of 1.606 mg/L, it is mainly caused by the desorption of organic hydrocarbon substances adsorbed in the sediments under certain conditions [15].

### GC/MS analysis

The petroleum contaminants in Taihu Lake were analyzed by GC/MS. The qualitative analysis showed that toxic organic pollutants in the water body were high, and the content of phthalic acid esters was the highest among all the organic pollutants. The organic pollutants included ethyl benzene, phthalic acid esters and PAHs which are belonging to the US EPA priority pollutants, and the toxic organic compounds included ethyl benzene, xylene, naphthalene, fluoranthene, benzo fluoranthene, benzo(a)pyrene, benzo(g,h,i)perylene belonging to China’s "preferentially controlled pollutants in water". These petroleum toxic substances have a relative long-term environmental impact, and generally are difficult to be degraded by micro-organisms in water, while easy to be absorbed by organisms. These toxic substances can gradually gather in the living bodies through the process of food chain, thus causing harm to human health.

**Figure 2:**
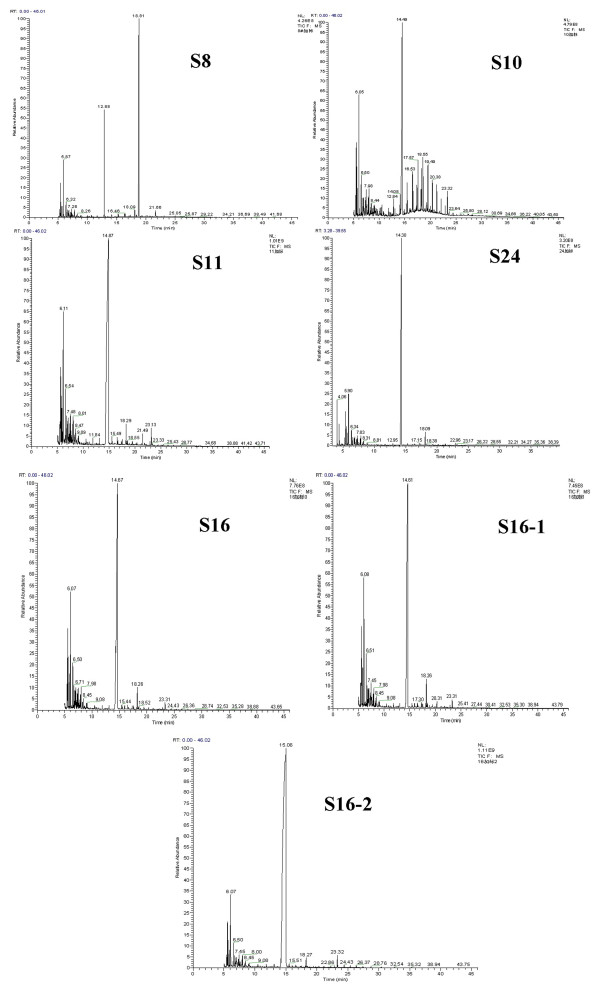
**The total ion chromatograms of the water samples from different sampling points.** The total ion chromatograms of the water samples obtained by GC/MS from S8, S10, S11, S24, S16, S16-1 and S16-2 are shown in Figure 2.

The chromatograms of the water samples obtained by GC/MS are shown in Figure 2. The compositions and relative contents of petroleum contaminants in the water samples were calculated using the square peaks normalizaion of the intergral regions.

**Figure 3:**
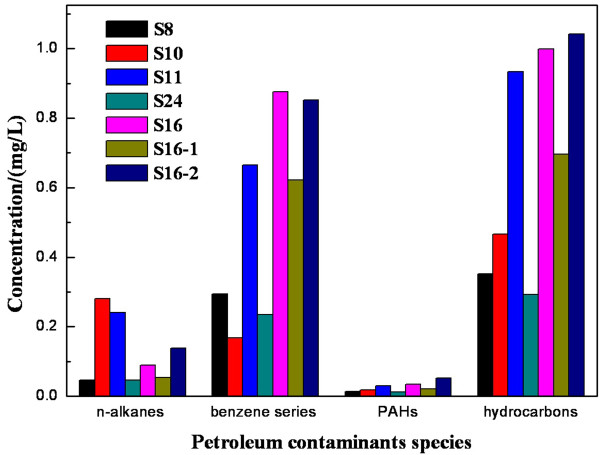
**The concentrations of petroleum-type pollutants in the water samples collected in different regions.** The concentrations of petroleum-type pollutants in the water samples from S8, S10, S11, S24, S16, S16-1 and S16-2 is shown in Figure 3, petroleum contaminants species shown in Figure 3 include n-alkanes, benzene series, PAHs and hydrocarbons.

The results of quantitative analysis of water samples in Taihu are shown in Figure 3. It can be seen that the content of benzene series compounds in oil contaminants was the highest, followed by n-alkanes and PAHs was the least, and the concentrations of the three ranged from 0.168 to 0.875 mg/L, 0.045 to 0.281 mg/L and 0.011 to 0.034 mg/L, respectively.

#### Distribution characteristics of n-alkanes

**Figure 4:**
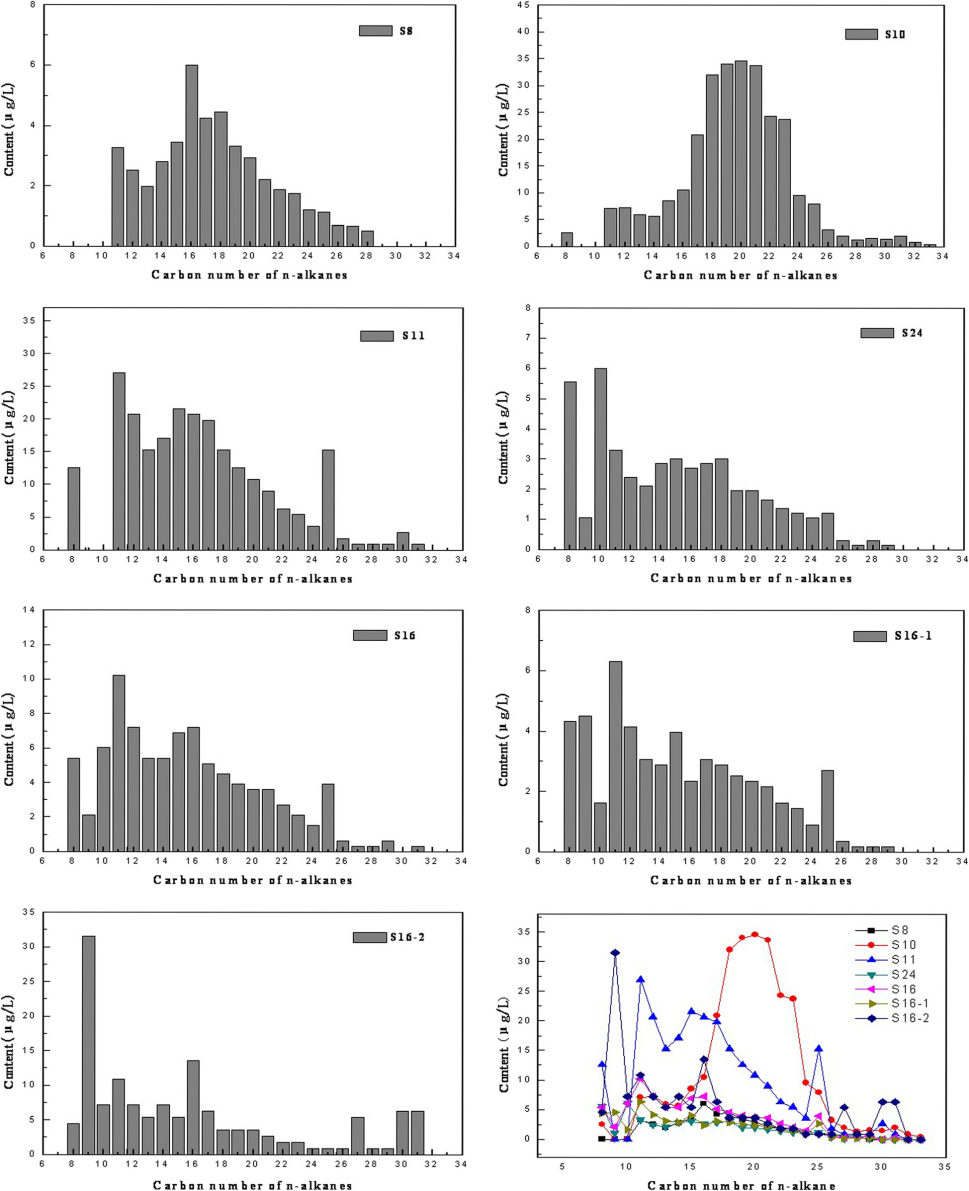
**The compositions of n-alkanes in the different water samples from Taihu Lake.** The compositions of n-alkanes in water samples from S8, S10, S11, S24, S16, S16-1 and S16-2 are shown in Figure 4, and the carbon number of n-alkane in each water sample is also shown in Figure 4.

The total contents of n-alkanes in Taihu water were in the following order: “Dapu”>“Xiaomeikou”>“Zhushan Bay”>“Qidu”>“Central Lake” and the results are presented in Figure 4. N-alkanes are the main component of petroleum hydrocarbons, and they are usually regarded as a classic organic geochemistry indices to distinguish biology, geological origin and artificial pollution [16].

Figure 4 shows the composition distribution characteristics of n-alkanes in Taihu Lake water. The content of n-alkanes on the bottom layer is the highest, followed by that on the surface layer and that on the middle layer is the lowest. Their mass concentrations were 0.139 mg/L, 0.089 mg/L and 0.054 mg/L respectively. The carbon number distribution of n-alkanes in Taihu Lake water ranged from C8 to C33. The composition distribution of n-alkanes in water ranged between 0.360-34.560 μg/L in sampling site S10, 0.900-21.600 μg/L in S11, 0.297-10.197 μg/L in S16, 0.150-6.000 μg/L in S24 and 0.525-6.000 μg/L in S8, respectively.

Relevant research showed that the n-alkanes derived from the oil, automobile exhaust and fossil fuel combustion are mainly low carbon aliphatic hydrocarbons, the dominant carbon number is C20 or C21 and the carbon number distribution displayed single peak distribution without odd-even predominance (OEP) [17]. The n-alkanes derived from terrestrial higher plants have long carbon chains (C27-C33), the carbon number distribution usually was single-peak state with significant advantage of odd carbon and it mainly takes C27, C29 or C31 as main peaks [18]. The n-alkanes derived from the low-level organisms such as algae, plankton and bacterial have relatively short carbon chains and the main peaks are C15, C17 or C19. The carbon number distribution showed a single-peak pattern without obvious odd-even predominance and high carbon numbers (>C25), and the bimodal distribution is considered to be mixed sources.

We can infer the sources of n-alkanes in water based on different characteristics [19]. Figure 4 illustrates that the distributions of n-alkanes showed a single-peak pattern without OEP, which is characteristic for the type of crude oil pollution [20]. In the bottom lake water of Zhushan Bay (S16), n-alkanes showed a bimodal distribution, indicating that in addition to the input from pollution sources of petroleum hydrocarbon, there is also input from natural organisms.

To further explore the source of n-alkanes in water, the molecular characteristic indicators of n-alkanes in Taihu Lake water were investigated and the results are presented in Table 2.

**Table 2:** Evaluation indices of n-alkanes for water from Lake Taihu

**Sampling points**	**Main peak carbon**	**Carbon number distribution**	**CPI**	**OEP**	**Pr/Ph**	**Pr/C17**	**Ph/C18**
**S8**	16	11-28	0.96	0.71	0.54	0.73	1.27
**S10**	20	8-33	1.11	1.03	0.60	0.54	0.59
**S11**	15,25	8-30	1.14	1.09	0.87	0.59	0.88
**S24**	18	8-29	0.68	0.85	1.29	0.47	0.35
**S16**	16	8-31	1.00	0.90	1.00	0.53	0.60
**S16-1**	15	8-29	1.27	1.43	0.91	0.59	0.69
**S16-2**	16	8-31	1.41	0.51	1.00	0.43	0.75

Note:

CPI: Carbon predominance index; OEP: Odd-even predominance; Pr: Phytane; Ph: Pristane.

The evaluation indices of n-alkanes include main peak carbon, carbon number distribution, CPI, OEP, Pr/Ph, Pr/C17, Ph/C18, from these indices, the source of n-alkanes in Taihu Lake water can be explored.

The main peak carbon number (Cmax) can be used to determine the source of n-alkanes [21]. Cmax is generally taken as a sign of the source and maturity of organic substances: Cmax is lower than C20 in organic substances with high maturity while higher than C25 in organic substances with low maturity. The fossil fuels (coal, oil, etc.) used by human generally have high maturity and the n-alkanes from the fossil fuels have a relatively low Cmax, while n-alkanes in higher plant are characterized by high Cmax. The main peak carbon number of n-alkanes in Taihu Lake water is relatively low, so the sources of alkanes are from high-maturity oil and its product as pollutants.

Carbon predominance index (CPI) can be used to indicate the relationship between biogenic origin and anthropogenic origin [22]. Generally speaking, the CPI value of human origin is close to 1, while most of the CPI values with plant origin are larger than 3 [23]. The CPI values of n-alkanes in Taihu Lake water ranged from 0.68 to 1.27, indicating that the anthropogenic activities is the major causes of petroleum hydrocarbon pollution in Taihu Lake.

Pristane (Pr) and phytane (Ph) are also important indicators of petroleum hydrocarbon contamination. The Pr/Ph ratio between 40 and 70 is the characteristic indication of biogenic hydrocarbon contamination; when the Pr/Ph is in the range of 1 to 3, to a certain degree, it reflects the man-made pollution of oil and its products [24]. When the values of Pr/C17 and Ph/C18 are relatively small, it is mainly from anthropogenic sources [25]. The ratios of Pr/Ph in this study range from 0.54 to 1.29, the ratios of Pr/C17 and Ph/C18 range from 0.43 to 0.73 and 0.35-1.27 respectively, and the ratios are all relatively small (Table 2), which further indicates existence of petroleum hydrocarbon contamination.

Based on the above analysis, the petroleum hydrocarbon contamination in Taihu Lake is mainly the result of human pollution, which is very serious and will last a long time. It is necessary to carry out well-designed management and control measures on pollution in Taihu Lake water according to different degrees of pollution in different regions.

#### Distribution characteristics of BTEXs

**Figure 5:**
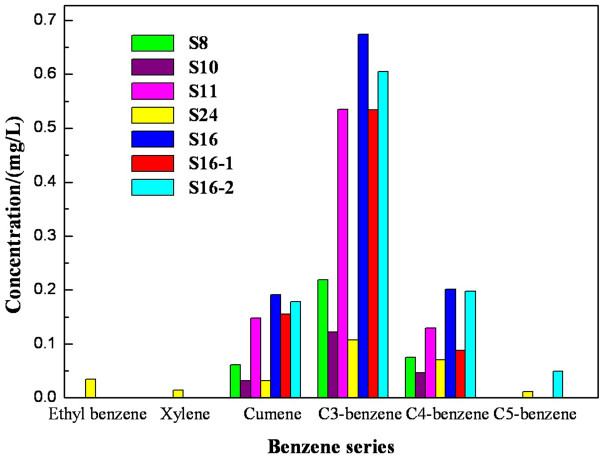
**The compositions of BTEXs in the different water samples.** The compositions of BTEXs in water samples from S8, S10, S11, S24, S16, S16-1 and S16-2 are shown in Figure 5. Benzene series shown in Figure 3 include ethyl benzene, xlyene, cumene, C3- benzene, C4-benzene and C5-benzene.

Benzene series (BTEXs) are mainly from the waste water discharged by petrochemicals, organic chemicals, coking chemical industry and so on. The priority controlled pollutants of BTEXs in the water environment include benzene, toluene, ethyl benzene, xylene, m-xylene, o-xylene, cumene and styrene. The total contents of benzene series in oil pollutants is in the order of S16>S11>S8>S24>S10. The contents sequence of benzene series in different levels of the same area is: the surface water(S16)>the bottom layer (S16-2)>the middle the lake (S16-2) (Figure 5).

“Standard Limits for Specific Items in Water head Area of Surface Water in Centralized Drinking Water” [14] stipulates that the concentrations of benzene, toluene, ethyl benzene, xylene, cumene and styrene should not exceed 0.01 mg/L, 0.7 mg/L, 0.3 mg/L, 0.5 mg/L, 0.25 mg/L and 0.02 mg/L, respectively. As shown in. Figure 5, benzene, toluene and styrene have not been detected in oil pollutants of Taihu Lake, and the detected benzene series were mainly C3-benzene, C4-benzene. The content of cumene ranged from 0.032 to 0.191 mg/L which were all lower than the standard limits. The concentration of xylene was in the range of not detected (ND~0.014 mg/L) It was also much smaller than the environmental standard limits.

#### Distribution characteristics of PAHs

PAHs are the organic compounds widely distributed in river and lake environment, which mainly come from the pollution brought by human activities and energy use (such as oil source, the incomplete combustion of fossil fuels). The total content of PAHs detected in Taihu Lake water ranged from 11.461 to 33.924 μg/L with the average value of 20.934 μg/L. The distribution characteristics of PAHs are shown in Table 3.

**Table 3:** Species and content of PAHs in different water samples (unit: μg/L)

**PAH**^**a**^	**S8**	**S10**	**S11**	**S24**	**S16**	**S16-1**	**S16-2**
**NaP**	4.075	3.899	8.240	2.715	11.961	8.498	16.480
**MNa**	6.016	5.128	8.240	2.060	7.310	4.378	10.300
**DMNa**	-^b^	6.599	6.523	2.435	11.629	4.635	6.850
**TMNa**	-	-	-	-	-	-	5.562
**AcP**	0.284	0.368	0.343	0.094	0.332	0.258	1.030
**Methyl biphenyl**	-	0.294	-	-	-	-	-
**Phe**	-	0.147	0.343	0.468	0.831	0.515	4.120
**MPhe**	1.716	0.368	1.717	1.217	0.598	1.030	1.133
**DMPhe**	-	-	0.721	1.966	0.764	1.030	2.060
**Flu**	-	0.066	-	-	0.199	0.335	1.030
**FL**	0.149	0.294	1.030	0.187	0.066	0.077	0.927
**Pyr**	0.448	0.221	0.687	0.159	0.033	0.052	0.618
**BaA**	-	-	0.343	0.094	0.033	0.026	0.206
**Chr**	-	-	0.412	0.066	0.133	0.052	0.618
**BbFL**	-	0.007	-	-	0.033	-	0.618
**BaP**	-	0.066	-	-	-	-	0.082
**BghiP**	-	-	-	-	-	0.026	0.041
**DBA**	-	0.037	-	-	-	-	0.072
**Total**	12.688	17.495	28.600	11.461	33.924	20.912	51.747

Note:

a NaP: naphthalene, MNa: methylnaphthalene, DMNa: Dimethylnaphthalene, TMNa: trimethyl-NaP, AcP: acenaphthene, Phe: phenanthrene, MPhe: methylphenanthrene, Flu: fluorene, FL: fluoranthene, Pyr: pyrene, BaA: benz[a]anthracene, Chr: chrysene, BbFL: benzo[b]fluoranthene, BaP: benzo[a]pyrene, DBA: dibenzo[a,h]anthracene, BP: benzo[ghi]perylene.

b Not detected.

The species and contents of PAHs in different water samples are shown in Table 3.

Zhushan Bay is connected with the lake inlet of Taige Canal, Yin Village Bay and Caoqiao River which carried high-concentration pollutants discharged by the nearby industrial areas. So the maximum content of PAHs is at S16 among different areas of Taihu Lake water. The total content of PAHs at site S11 is about 28.600 μg/L which is followed by S10 and S8 (17.495 μg/L and 12.688 μg/L respectively), and the content of PAHs at S24 was the lowest (0.011 μg/L). The distribution feature of PAHs in different water levels is bottom layer>surface layer>middle layer. The PAHs on the bottom layer have the most varieties and the highest content. As a typical shallow lake, the content of PAHs in sediments of Taihu Lake has some effect on the bottom water body, and the distribution of PAHs in Taihu Lake water is roughly consistent to the trend of PAHs in surface layer sediments [26].

**Figure 6:**
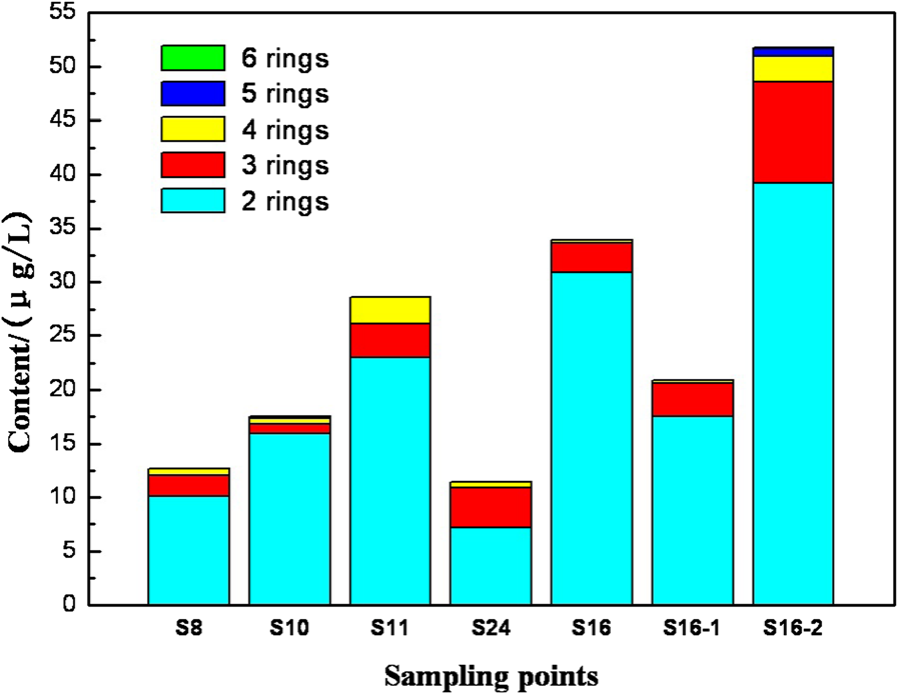
**Distribution of 2-, 3-, 4, 5-, 6-ring and total PAHs in different water samples.** The distribution of 2-, 3-, 4, 5-, 6-ring and the total contents of PAHs in water samples from S8, S10, S11, S24, S16, S16-1:and S16-2 are shown in Figure 6.

Figure 6 shows the distribution of 2-, 3-, 4, 5-, 6-ring and total PAHs in water. PAHs in the water of Taihu Lake are mainly low-ring aromatic hydrocarbons with 2 rings or 3 rings, while the high-ring aromatic hydrocarbons with 4 to 6 rings are relatively few. The upper limit value of benzo(a)pyrene is 2.80 ng/L stipulated in China’s Surface Water Environmental Standard. In the various sampling sites of Taihu Lake, only at site S16 and S10 were detected to have benzo(a)pyrene whose content exceeded the limited value.

PAHs from different sources have different types and different distribution characteristics. The man-made sources mainly refer to oil pollution and combustion source, and it is difficult to completely distinguish them in the environment. At present, the two sources of PAHs are mainly distinguished by utilizing characteristic compound index, including the ratios of Phe/Ant, Chr/BaA, Flu/Pyr, Flu/(Flu + Pyr), IP/(IP + BghiP), LMW/HMW, etc. [27].

Typically, low molecular weight PAHs with 2 to 3 rings are from oil contamination, while high molecular weight PAHs with 4 to 6 rings are from the pyrolysis of fossil fuels [28]. Table 4 shows the evaluation indices of PAHs for the water from Lake Taihu. The Phe/Ant ratios are larger than 15 and the LMW/HMW (the proportion of 2-ring and 3-ring PAHs to 4-ring PAHs) in Taihu Lake water is relatively high, which indicates that the PAHs in Taihu Lake water mainly came from oil pollution, it is reasonable to relate this to the busy water transport system in this region. The results of Chr/BaA and Flu/(Flu + Pyr) ratios showed a mixture of petroleum and combustion contamination.

**Table 4:** Evaluation indices of PAHs for the water from Lake Taihu

**Index**	**Combustion sources**	**Sources of oil pollution**	**Lake water**
**Phe/Ant**^**a**^	Less than 10	Greater than 15	Greater than 15
**Chr/BaA**	less than 1	Greater than 1	0.70-4.03
**Flu/(Flu + Pyr)**	Greater than 0.4	Less than 0.4	0.25-0.67
**IP/(IP + BghiP)**	Less than 0.2	Less than 0.2	-^**b**^
**LMW/HMW**	Low	High	10.57-112.84

Note:

a: Phe: Phenanthrene; Ant: Anthracene; Chr: Chrysene; BaA: Benz[a]anthracene; Flu: fluoranthene; Pyr: pyrene; IP: Indenoindenes; BghiP: Benzo[ghi]perylene;

LMW/HMW: The ratio of 2-ring and 3-ring PAHs to 4-ring PAHs.

b: Not detected.

The evaluation indices of PAHs in Lake Taihu are shown in Table 4, these indices include Phe/Ant, Chr/BaA, Flu/(Flu + Pyr), IP/(IP + BghiP) and LMW/HMW, from which the sources of PAHs can be distinguished

The petroleum pollutants have many ways of migration in the water and they are easy to spread and transfer. However, natural factors have very limited degradation effect on the oil pollutants, and the oil pollutants are difficult to do self-purification in the environment, thus resulting in accumulation of pollution which is difficult to recover [29]. In the process of standardization and management of surface water quality, the key to control oil pollution is mainly to prevent it; to reasonably control the oil content of the rivers into the lake will help reduce oil pollution in Taihu Lake. As oil pollution is relatively serious at the bottom of the water body, to regularly dredge the bottom sediment in Taihu Lake is also conducive to the control of oil pollution in Taihu Lake.

## Conclusion

The water pollution was relatively serious in the river entrance into the lake, and the content of petroleum pollutants exceeded the limit value of oil Class IV in Surface Water Quality Standards [14]. According to the sources apportionment of n-alkanes and PAHs, it can be concluded that the n-alkanes pollution was mainly the result of human pollution and the PAHs contamination in Taihu Lake was the result of both petroleum pollution and combustion.

The petroleum contamination in Taihu Lake water was mainly from oil discharge and the combustion of fossil fuel such as coal, oil and others. For the purpose of improving the water quality of Taihu Lake, we should vigorously promote the scientific treatment of oily wastewater, strengthen the management of lake pollution, focus on the control over pollution sources such as the industrial and household sewage, oily waste water from the vessels and so on, and timely dredge the sediment in the seriously polluted areas.

## Abbreviations

AcP: Acenaphthene; Ant: Anthracene; BaA: Benz[a]anthracene; BaP: Benzo[a]pyrene; BbFL: Benzo[b]fluoranthene; BP: Benzo[ghi]perylene; Chr: Chrysene; Cmax: Main peak carbon number; CPI: Carbon predominance index; DBA: Dibenzo[a,h]anthracene; DMNa: Dimethylnaphthalene; FL: Fuoranthene; Flu: Fluoranthene; GC/MS: Gas Chromatography and Mass Spectrometry; IP: Indenoindenes; LMW/HMW: The ratio of 2-ring and 3-ring PAHs to 4-ring PAHs; MNa: Methylnaphthalene; MPhe: Methylphenanthrene; NaP: Naphthalene; OEP: Odd-even predominance; PAHs: Polycyclic aromatic hydrocarbons; Ph: Phytane; Phe: Phenanthrene; Pr: Pristane; Pyr: Pyrene; TMNa: Trimethyl-NaP.

## Competing interests

The authors declare that they have no completing interests.

## Authors’ contributions

All authors designed the study. All authors contributed to data ananlysis and to finalizing the manuscript, all authors have read and approved the final version.
